# Supplementary material for: Drug Resistance in Medulloblastoma Is Driven by YB-1, ABCB1 and a Seven-Gene Drug Signature
Source: Cancers (Basel). 2023 Feb 8;15(4):1086. doi: 10.3390/cancers15041086 (PMC9954169; doi:10.3390/cancers15041086)
Supplement: Supplementary file 1 [file cancers-15-01086-s001.zip › Supplementary Methods File SB.pdf]

Supplementary Methods File SB

Zymography

Snap frozen supernatants were thawed on ice and then directly mixed 1:4 with 4 X β-mercaptoethanol-free SDS-PAGE sample buffer and vortexed for 5 seconds before being added directly to 10% w/v polyacrylamide gels in which the resolving gel was supplemented with 1 mg/ml gelatine (Sigma). Following electrophoresis, the gels were incubated for one hour at room temperature with gentle agitation in 2.5% v/v Triton X-100. This renaturing buffer was replaced with developing buffer (BioRad #161076) and incubated with the gels for 60 minutes at room temperature, followed by an overnight incubation at 37 °C with fresh developing buffer. Following several washes with dH_2O, gels were stained in 40% v/v methanol, 10% v/v glacial acetic acid, 0.5% w/v Commassie blue R-250 at room temperature for 15 minutes, destained (40% v/v methanol, 10% v/v glacial acetic acid) for two hours or until white bands started to appear on the gels, and fixed (5% w/v glycerine, 30% v/v methanol) for 30 minutes prior to imaging. The intensity of the bands was evaluated with ImageJ software to give an indication of the level of secreted MMP-2 and MMP-9.
