# Supplementary material for: Drug Resistance in Medulloblastoma Is Driven by YB-1, ABCB1 and a Seven-Gene Drug Signature
Source: Cancers (Basel). 2023 Feb 8;15(4):1086. doi: 10.3390/cancers15041086 (PMC9954169; doi:10.3390/cancers15041086)
Supplement: Supplementary file 1 [file cancers-15-01086-s001.zip › Supplementary Results File SA.pdf]

Supplementary Results File SA

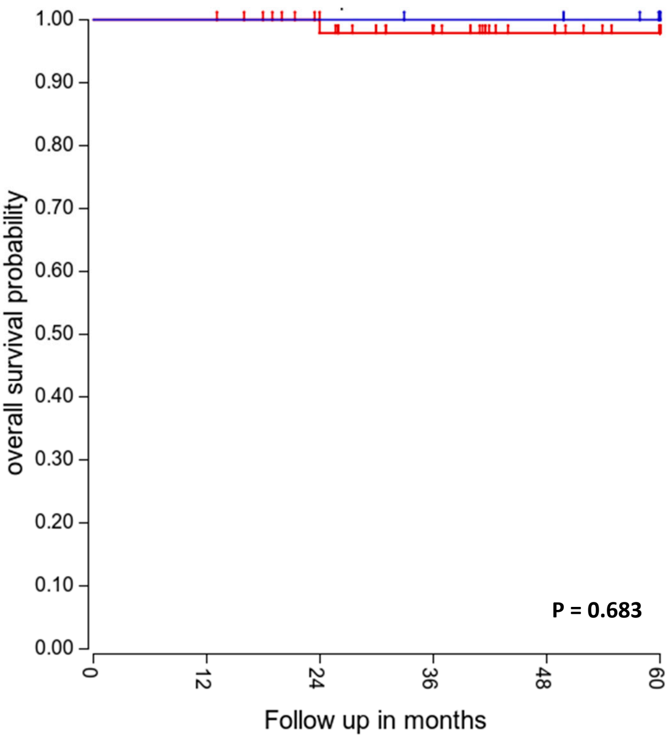

Supplementary Figure S1. YB-1 gene expression does not correlate with survival outcomes in WNT medulloblastoma. R2 genomics analysis (http://r2.amc.nl) was employed to explore correlation between YB-1 gene expression and survival in WNT subgroup medulloblastoma. Kaplan Meier analysis showed no association between YB-1 expression and 5 year overall survival probability in patients with WNT medulloblastoma (P = 0.683). N = 70 [1]. Survival curves compared using the Log-rank (Mantel-Cox) test.

A

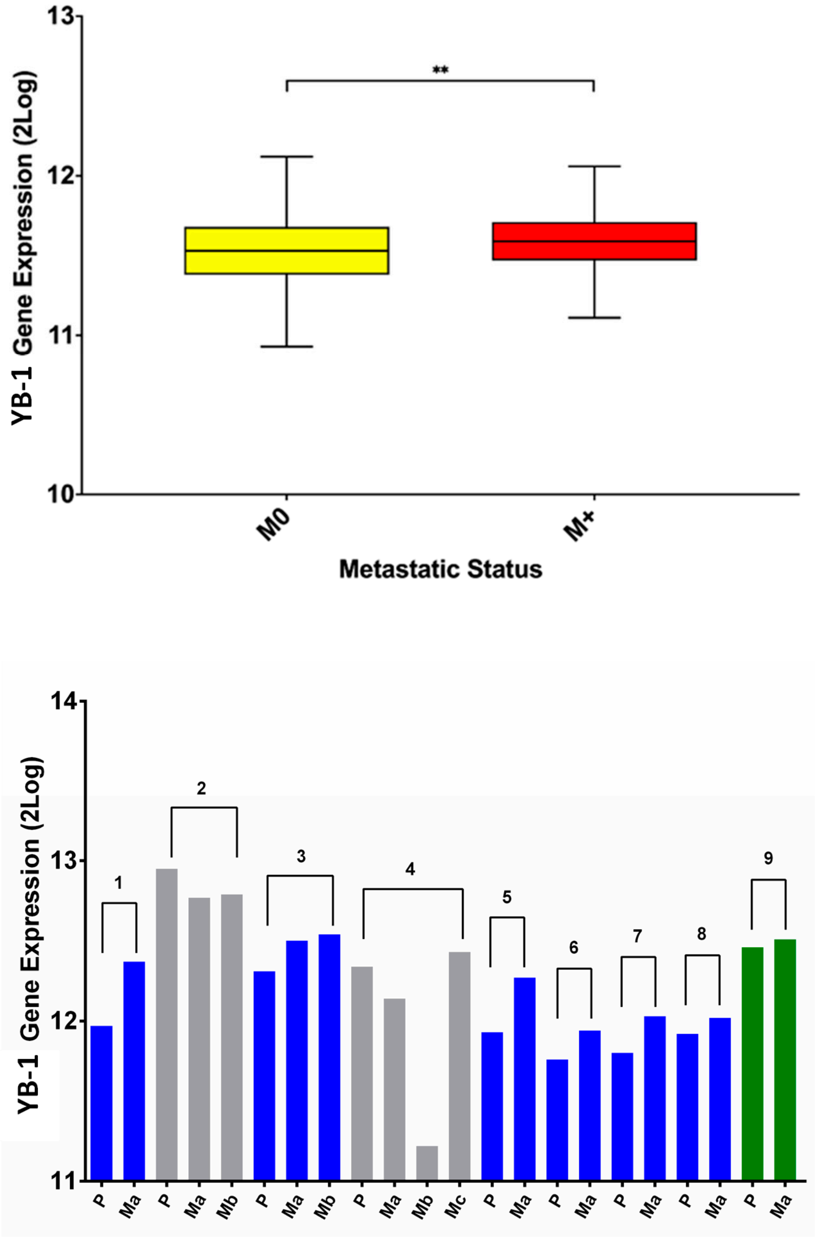

Supplementary Figure S2. YB-1 gene expression is elevated in metastatic tumours. A) YB-1 gene expression was found to be significantly higher in metastatic tumours (M+; n = 176) compared to non-metastatic tumours (M0; n = 397); **P < 0.01 [1]. Significance analysed by unpaired t-test with Welch's correction. B) YB-1 expression was frequently elevated in metastatic tumours when compared with the appropriate primary pair. N = 22 [2]. P represents primary tumour and Ma/b/c represent first/second/third metastases. Brackets define the samples attributed to each patient. SHH-classified tumours are denoted in green, Group 3 tumours in grey and Group 4 tumours in blue.

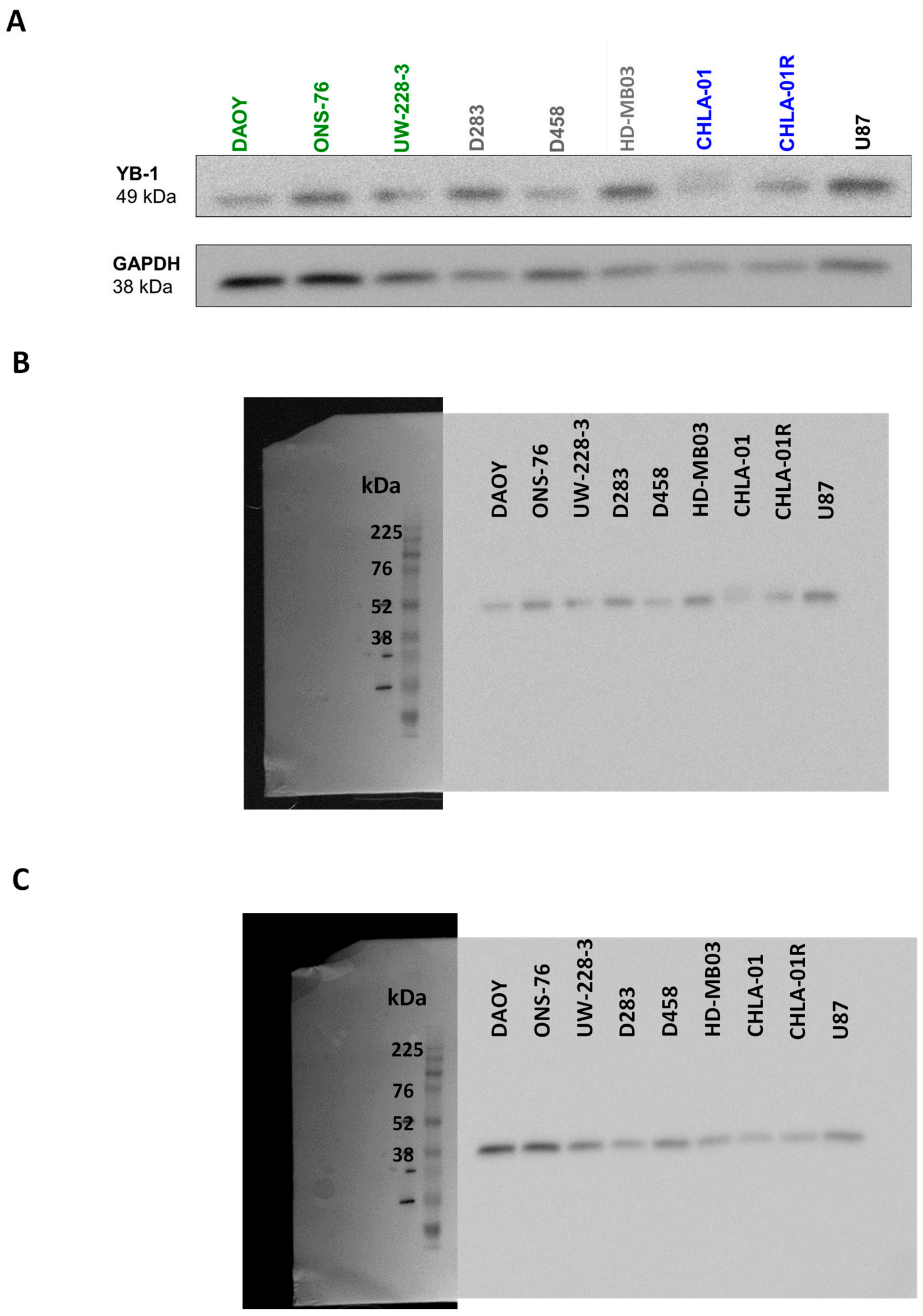

Supplementary Figure S3. YB-1 is expressed across a range of medulloblastoma cell lines. A) A representative western blot for total YB-1 expression across SHH, Group 3 and Group 4 established medulloblastoma cell lines. GAPDH served as a loading control. B) Full length western blot for YB-1 expression (49 kDa). C) Full length western blot for GAPDH expression (36 kDa). Protein expression was visualised and quantified by chemiluminescence imaging.

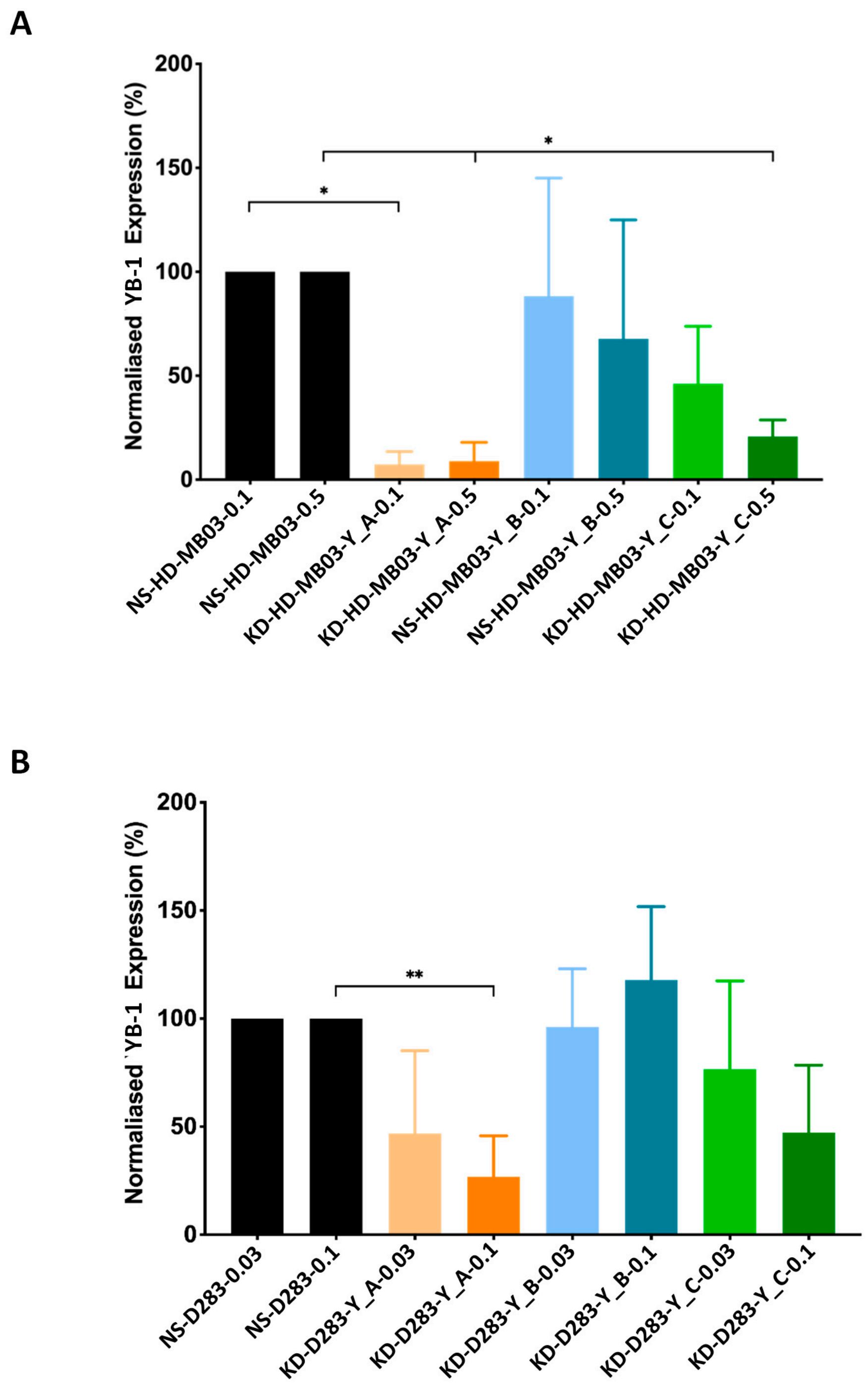

Supplementary Figure S4. shRNA-mediated knockdown of YB-1 effectively depletes YB-1 mRNA expression in HD-MB03 and D283 Group 3 medulloblastoma cell lines. A) In HD-MB03 cells, YB-1 mRNA was significantly depleted using the YB-1_A shRNA construct at a multiplicity of infection (MOI) of 0.1 and 0.5 (KD-HD-MB03-Y_A-0.1 and KD-HD-MB03-Y_A-0.5) and the YB-1_C construct at an MOI of 0.5 (KD-HD-MB03-Y_C-0.5), compared to the appropriate non-silencing control cell line (NS-HD-MB-03-Y_A-0.1 and NS-HD-MB03-Y_A-0.5). B) In D283 cells, YB-1 was significantly depleted using the YB-1_A shRNA construct at an MOI of 0.1 (KD-D283-Y_A-0.1) compared to the non-silencing control (NS-D283-Y_A-0.1). Accordingly, the YB-1_A construct at an MOI of 0.1 would be taken forward for further experiments. Relative YB-1 expression displayed as fold change ($2^{-\Delta\Delta Cq}$) relative to the appropriate non-silencing control. n = 3; mean ± SEM; **P < 0.01; *P < 0.05. Significance assessed by ordinary one-way ANOVA analysis with Sidak's multiple comparisons test.

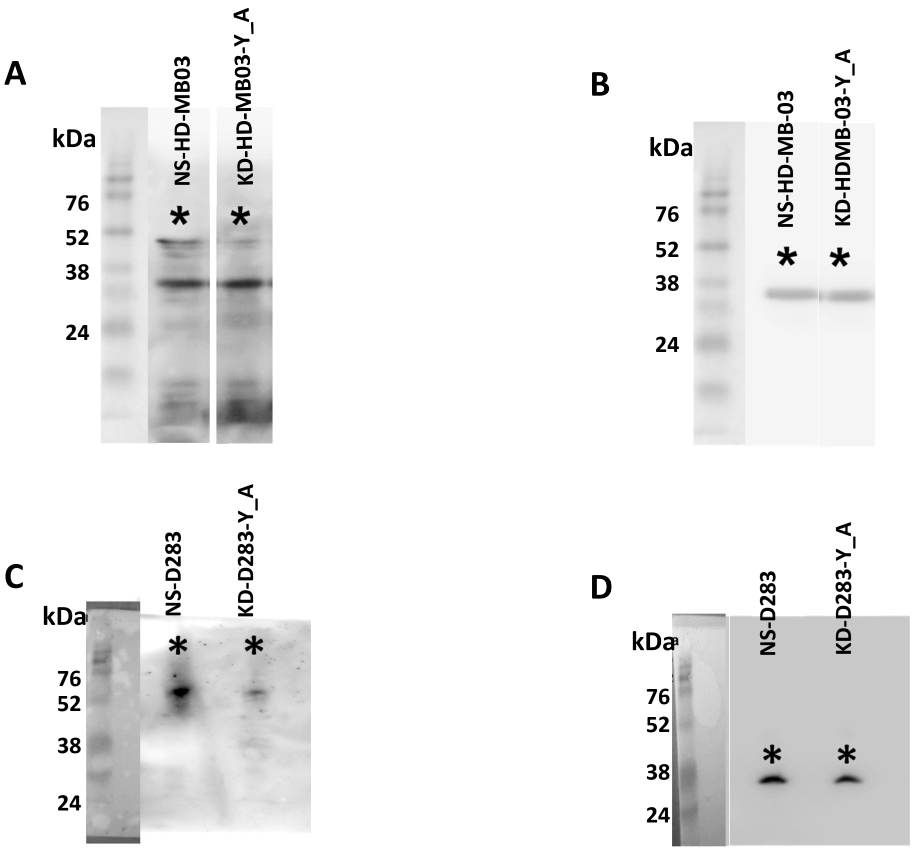

Supplementary Figure S5. shRNA-mediated knockdown of YB-1 mRNA effectively depletes YB-1 protein expression in HD-MB03 and D283 Group 3 medulloblastoma cell lines. A) Full western blot for total YB-1 expression across NS-HDMB-03 and KD-HD-MB03-Y_A cell lines (bands of interest marked by asterisk, 49 kDa; lower band represents GAPDH, visualised due to the use of a common secondary antibody). B) Full western blot for total GAPDH expression across NS-HD-MB03 and KD-HD-MB03-Y_A cell lines (bands of interest marked by asterisk; 36 kDa). C) Full western blot for total YB-1 expression across NS-D283 and KD-D283-Y_A cell lines (bands of interest marked by asterisk; 49 kDa). D) Full western blot for total GAPDH expression across NS-D283 and KD-D283-Y_A cell lines (bands of interest marked by asterisk; 36 kDa).

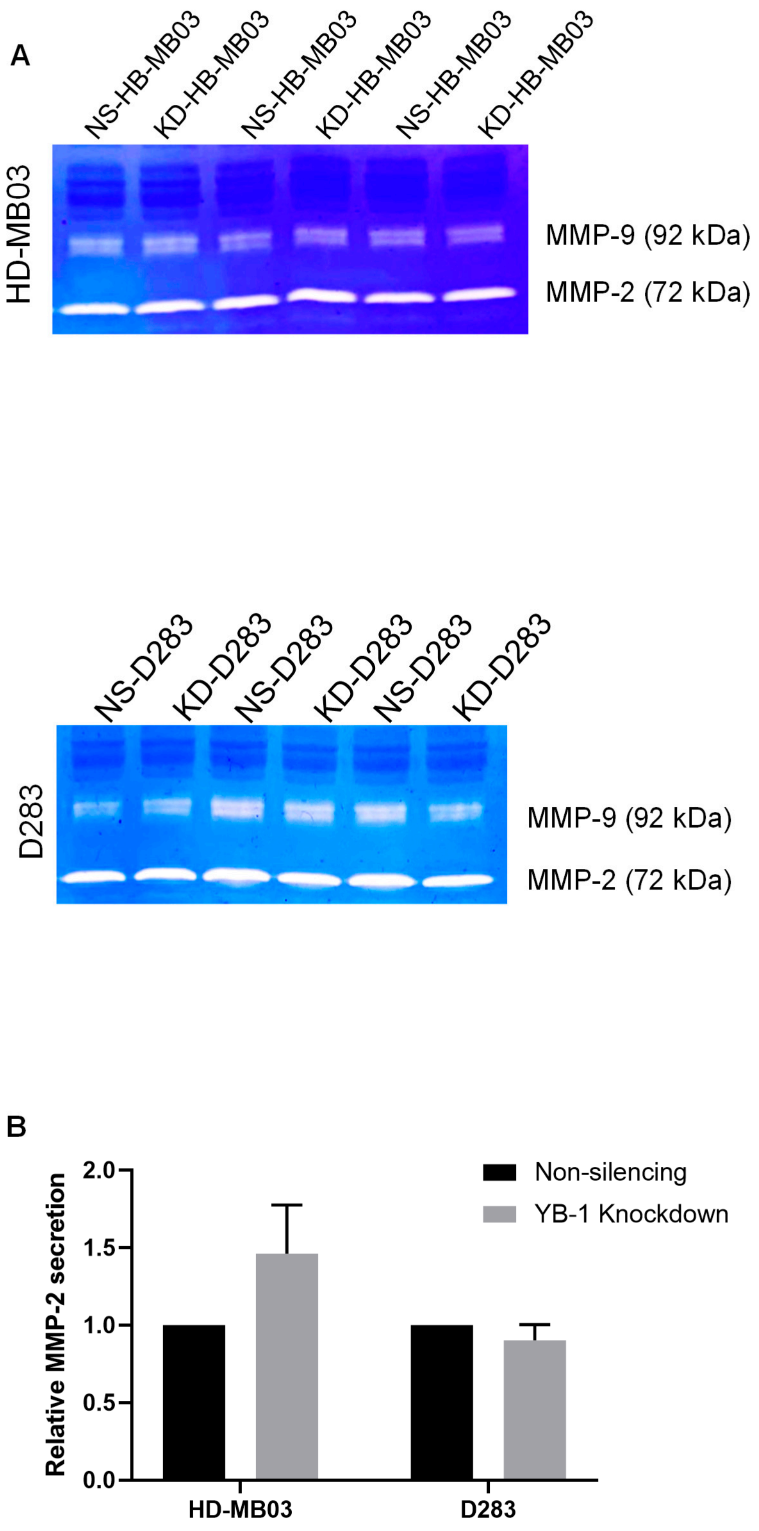

Supplementary Figure S6. Zymography analysis of MMP-2 and MMP-9 activity in HD-MB03 and D283 cell lines following YB-1 knockdown. A) Gelatin zymography of conditioned media collected from HD-MB03 and D283 YB-1-knockdown and non-silencing cell lines was undertaken. Representative blots shown. B) Densitometry analysis revealed no significant difference in MMP-2/-9 secretion as a result of YB-1 knockdown. Error bars represent the mean ± SEM of 3 independent experiments. Significance was assessed by Student's T-Test.

A

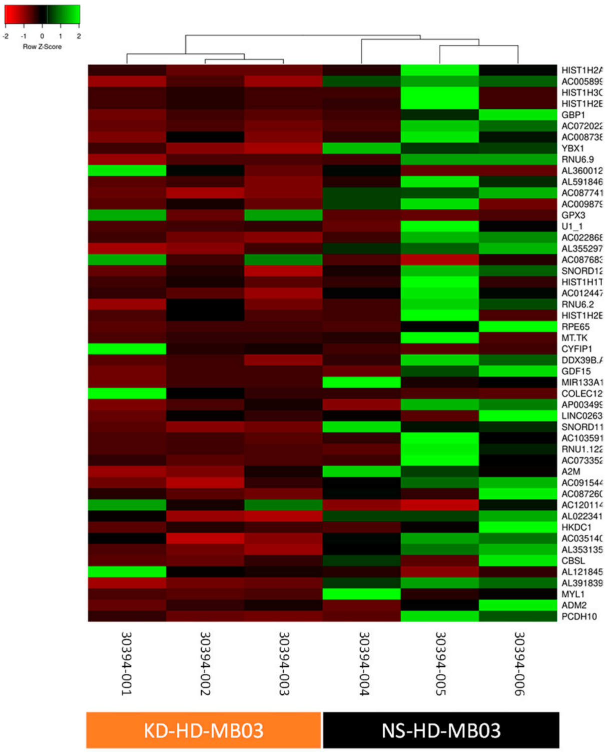

B

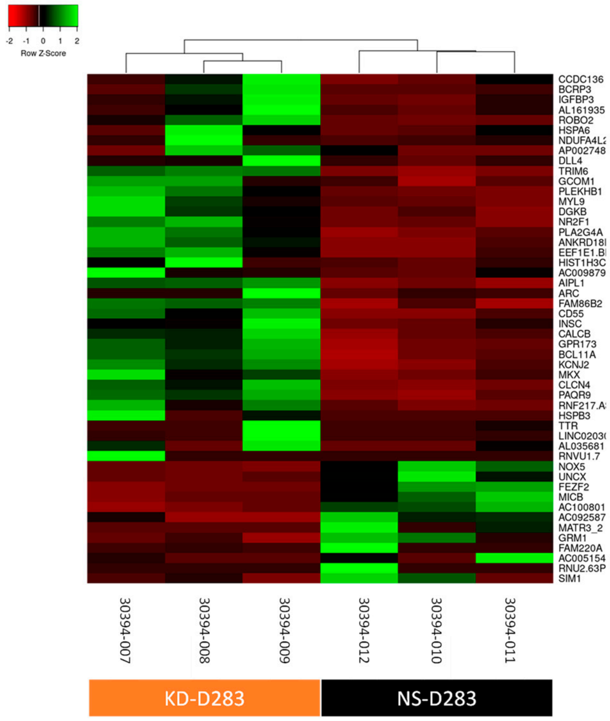

Supplementary Figure S7. Unsupervised clustering of YB-1-knockdown and non-silencing control HD-MB03 and D283 cell lines. Unsupervised transformed gene counts were used to create a heatmap representing the top 50 most altered genes across all knockdown and non-silencing samples. The colour scale signifies the relative expression level of a gene across all samples. Expression levels above the mean are shown as green and below the mean are red. Differences in gene expression between KD-HD-MB03 and NS-HD-MB03 (A) and KD-D283 and NS-D283 (B) were visible, with triplicates from each sample set clustering together.

A

Upstream Regulator	Activation z-score	p-value
BCR (complex)	-7.5	1.5E-08
LARP1	7.3	6.4E-19
CKAP2L	-6.1	4.6E-19
ERBB2	-5.9	2.2E-08
RABL6	-5.6	1.3E-10
TP53	5.5	1.3E-05
FOXM1	-5.3	9.0E-10
MYC	-5.3	4.5E-09
CD3E	-5.0	2.8E-09
DDX5	-5.0	1.4E-07

B

Category	Disease/Function Annotation	P-Value	Activation Z-Score
Cellular Development, Cellular Growth and Proliferation	Cell Proliferation of Tumour Cell Lines	8.00E-05	-9
Cell Death and Survival	Cell Survival	1.60E-10	-8.8
Cell Death and Survival	Cell Viability	3.50E-11	-8.5
Infectious Diseases, Organismal Injury and Abnormalities	Viral Infection	5.00E-03	-8.4
Infectious Diseases, Organismal Injury and Abnormalities	Cell Viability of Tumour Cell Lines	4.11E-11	-7.7
Infectious Diseases, Organismal Injury and Abnormalities	Infection of Cells	1.30E-03	-7.2
Infectious Diseases, Organismal Injury and Abnormalities	Infection by Retroviridae	1.10E-03	-6.9
Infectious Diseases, Organismal Injury and Abnormalities	HIV Infection	9.30E-04	-6.9
Infectious Diseases, Organismal Injury and Abnormalities	Infection by HIV-1	3.90E-04	-6.8
Cell Death and Survival	Cell Viability of Colorectal Cancer Cell Lines	4.30E-09	-6.2
Cellular Response to Therapeutics	Sensitivity of Tumour Cell Lines	6.20E-08	5.9

Supplementary Figure S8. Ingenuity Pathway Analysis (IPA) of human medulloblastoma patients with high/low YB-1 gene expression. Pathway analysis of significantly differentially expressed genes identified from a patient medulloblastoma cohort (n = 612) separated by high/low YB-1 expression was conducted using IPA software (Qiagen). A) Upstream regulator analysis was ranked by activation z-score. The top 10 most significantly dysregulated upstream regulators are shown. B) Molecular and Cellular functions were ranked by activation z-score and the top most dysregulated, significant functions presented. The patient dataset[1] was accessed by way of R2: Genomics and Visualisation Platform (http://r2.amc.nl) and the cut-off between high YB-1 expression and low YB-1 expression groups was selected by which p-values obtained from the log-rank test were minimized.

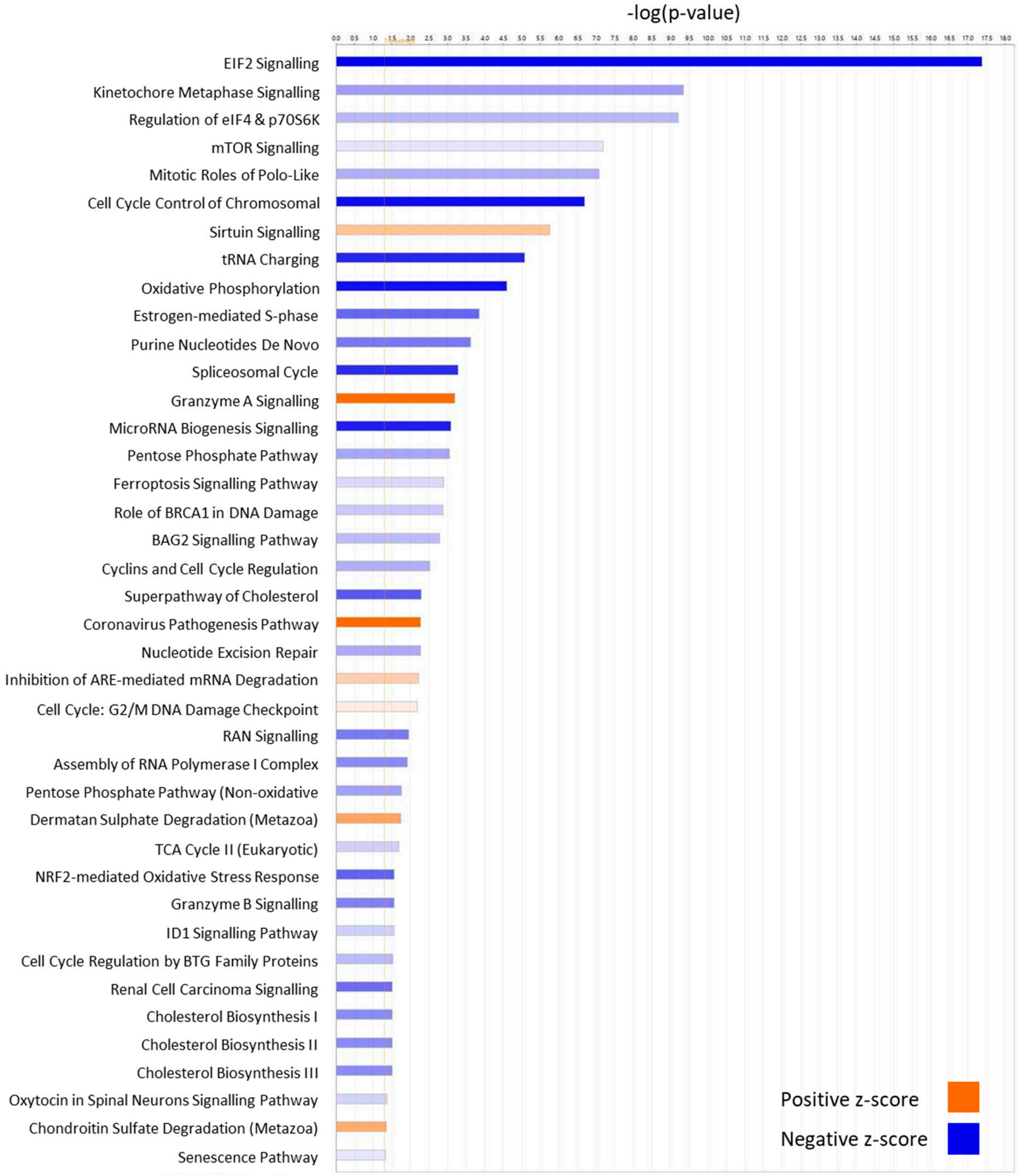

Supplementary Figure S9. Ingenuity Pathway Analysis (IPA) of a human medulloblastoma cohort. Pathway analysis of significantly differentially expressed genes identified from a patient medulloblastoma cohort (n = 612) separated by high/low YB-1 expression was conducted using IPA software (Qiagen). The results of canonical pathway analysis are presented. Only pathways from which a z-score could be calculated are shown. The patient dataset[1] was accessed by way of R2: Genomics and Visualisation Platform (http://r2.amc.nl) and the cut-off between high YB-1 expression and low YB-1 expression groups was selected by which p-values obtained from the log-rank test were minimized.

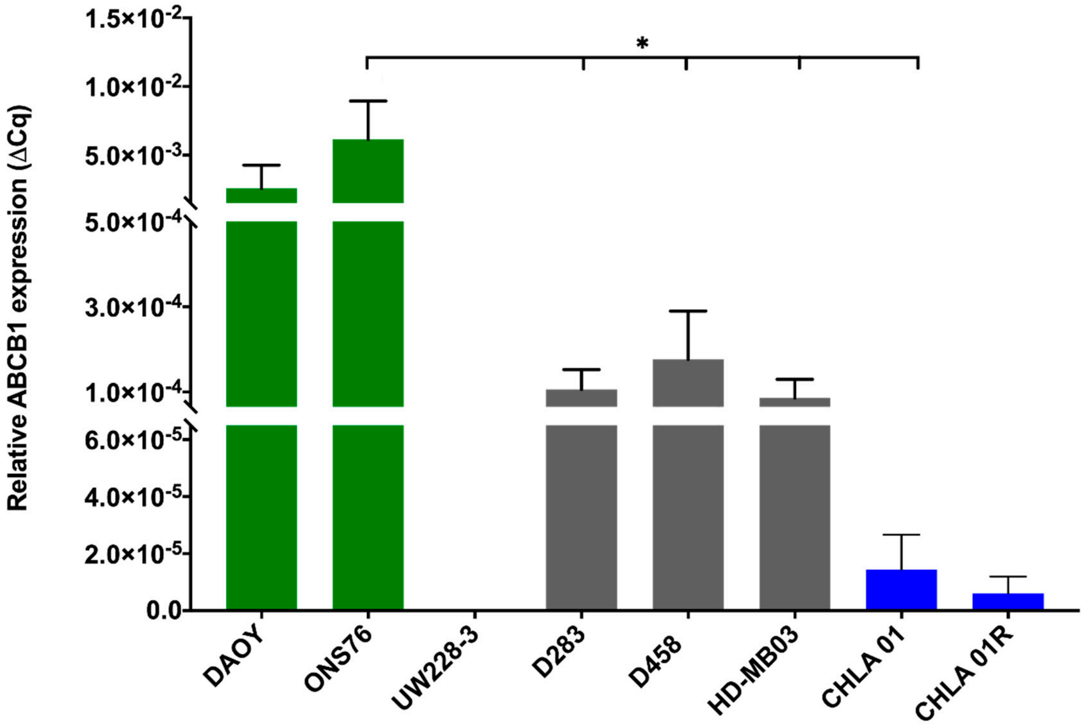

Supplementary Figure S10. *ABCB1* expression is highest in SHH cell lines. Analysis of *ABCB1* expression by qRT-PCR demonstrated that *ABCB1* was expressed across all available medulloblastoma cell lines apart from UW-228-3, with expression highest in SHH (green) and Group 3 (grey) sub-groups and lowest in Group 4 (blue) subgroups. Relative *ABCB1* expression ($2^{-\Delta Cq}$) was calculated with respect to housekeeping gene *GAPDH*. n = 3; Mean ± SEM; *P < 0.05. Significance was calculated by ordinary one-way ANOVA analysis with Tukey's multiple comparisons test.

A

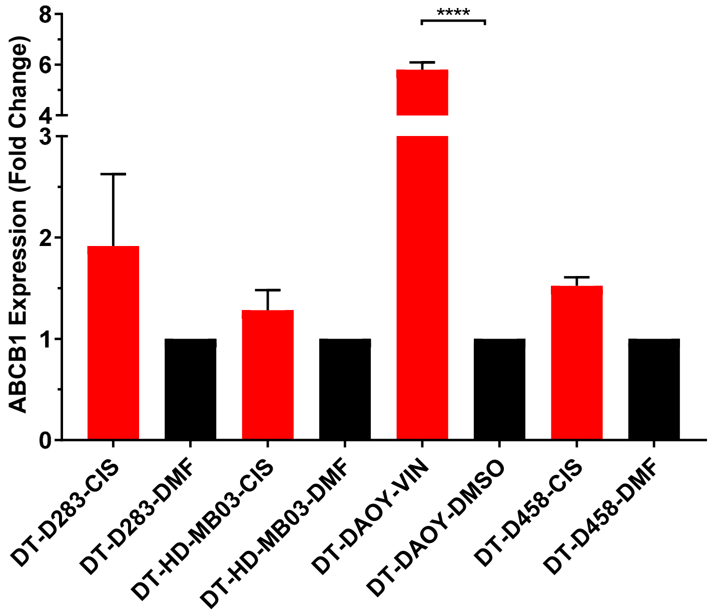

B

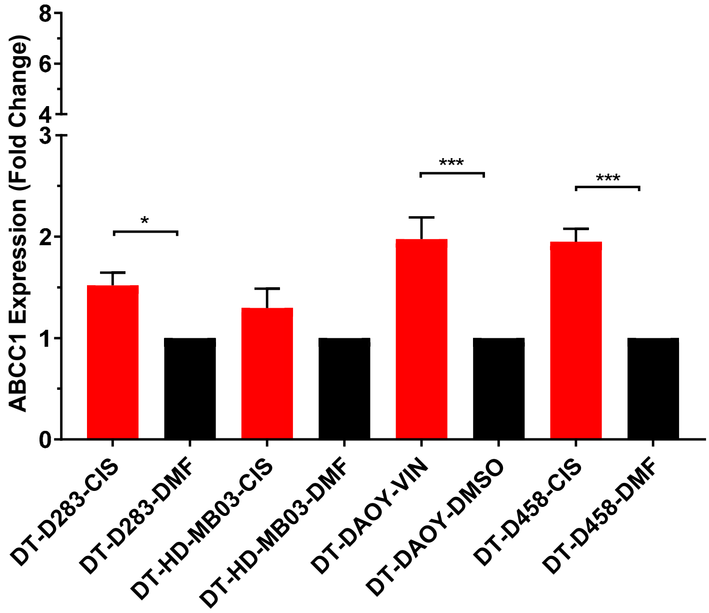

Supplementary Figure S11. ABC transporter expression is altered in drug-tolerant cell lines. A) Assessment of *ABCB1* expression across drug-tolerant cell lines revealed elevated expression in DT-DAOY-VIN cells compared to the DT-DAOY-DMSO line. B) Expression of *ABCC1* was found to be signficantly increased in DT-DAOY-VIN, DT-D458-CIS and DT-D283-CIS lines relative to appropriate vehicle treated controls. Fold change in ABC transporter gene expression was calculated relative to the *GAPDH* housekeeping gene and vehicle-treated control cell line ($2^{-\Delta\Delta Cq}$). N = 3. Mean ± SEM. Significance was assessed using ordinary one-way ANOVA analyses with Sidak's multiple comparisons test

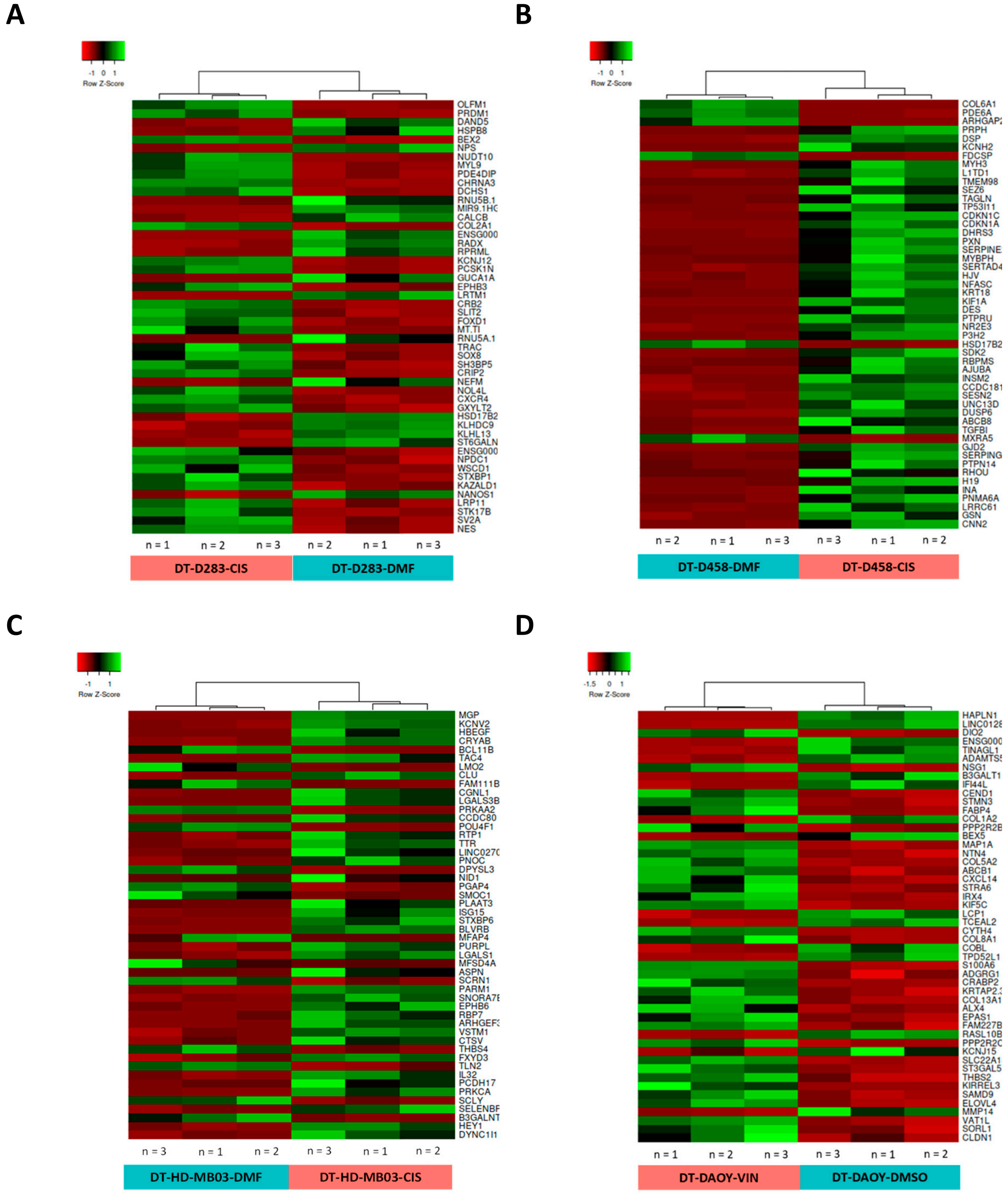

Supplementary Figure S12. Unsupervised clustering of drug-tolerant and sister vehicle-treated control cell lines. Unsupervised transformed gene counts were used to create heatmaps representing the top 50 genes with the largest variance across all D283 (A), D458 (B), HD-MB03 (C) and DAOY (D) drug-tolerant and vehicle-treated control samples. The colour scale signifies the relative expression level of a gene across all samples. Expression levels above the mean are shown as green and below the mean are red. Differences in gene expression between drug-tolerant and vehicle-treated cell lines were clear, with triplicates from each sample set clustering together.

Comparison	Fold-Resistance of DT cell line	Significantly up-regulated genes	Significantly down-regulated genes	Total Significantly DEGs
DT-D283-CIS v. DT-D283-DMF	2.5	694	600	1294
DT-D458-CIS v. DT-D458-DMF	18.5	1238	910	2148
DT-HD-MB03-CIS v. DT-HD-MB03-DMF	1.6	515	395	910
DT-DAOY-VIN v. DT-DAOY-DMSO	4	606	662	1268

Supplementary Figure S13. Differential gene expression profiles of drug-tolerant cell lines.

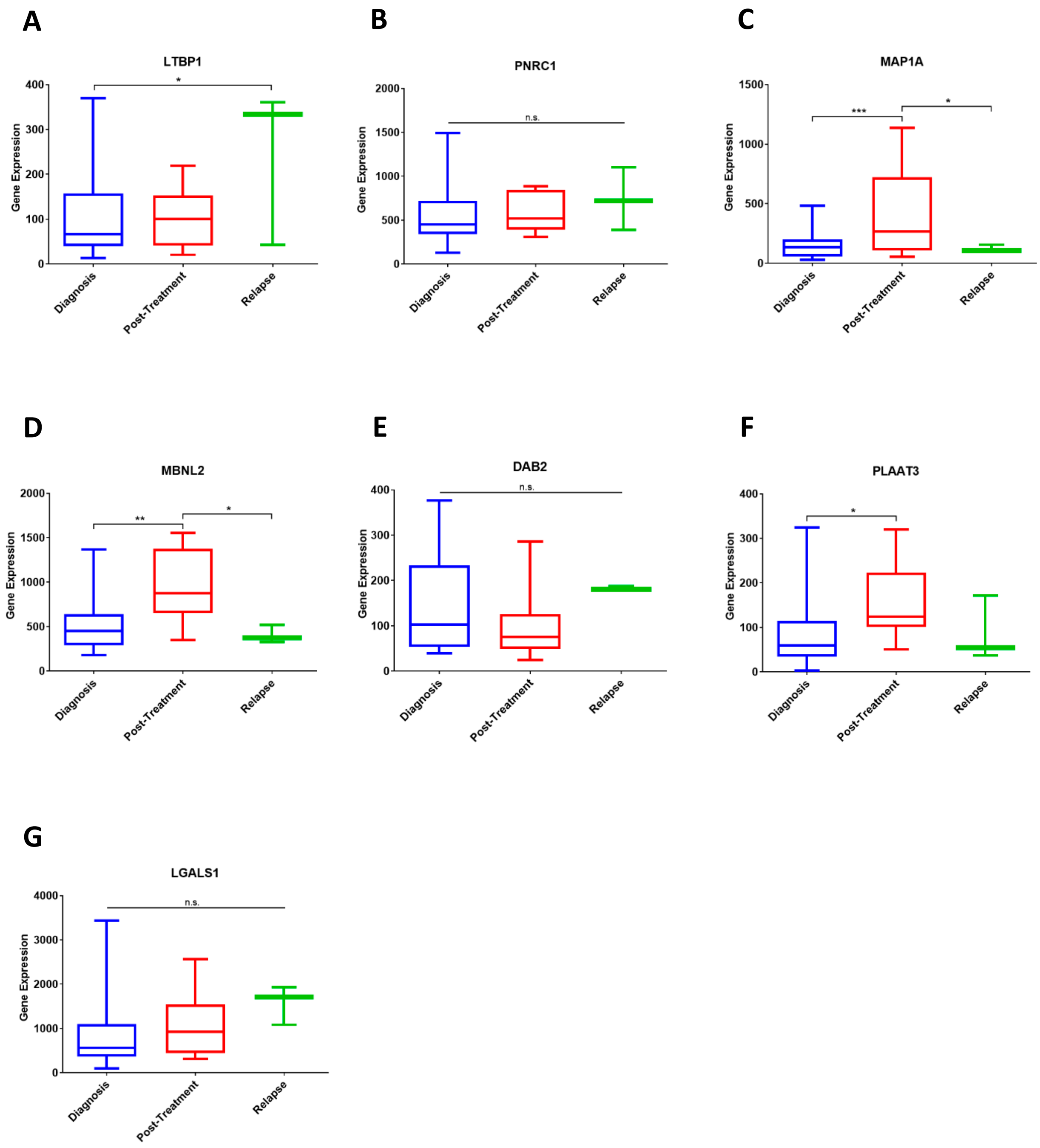

Supplementary Figure S14. Seven-gene drug tolerant signature expression in medulloblastoma diagnostic, post-treatment and relapse dataset. Gene expression of *LTBP1*, *PNRC1, MAP1A, MBNL2, DAB2, PLAAT3* and *LGALS1* was investigated in a small-scale medulloblastoma dataset containing diagnostic, post-treatment and relapse patient samples. Diagnosis n = 46; post-treatment n = 8, relapse n = 3. Expression displayed as box plots showing the sample minimum (lower line) and the sample maximum (upper line). Dataset (Tumor Medulloblastoma public - Delattre - 57 - MAS5.0 - u133p2) accessed using R2: Genomics Analysis and Visualization Platform[3]. *P < 0.05, **P < 0.01, ***P < 0.001, n.s. = not significant.

References

1 Cavalli FMG, Remke M, Rampasek L, Peacock J, Shih DJH, Luu Bet al (2017) Intertumoral Heterogeneity within Medulloblastoma Subgroups. Cancer Cell 31: 737-754.e736 Doi https://doi.org/10.1016/j.ccell.2017.05.005

2 Wang X, Dubuc AM, Ramaswamy V, Mack S, Gendoo DM, Remke Met al (2015) Medulloblastoma subgroups remain stable across primary and metastatic compartments. Acta Neuropathol 129: 449-457 Doi 10.1007/s00401-015-1389-0

3 Weishaupt H, Johansson P, Sundström A, Lubovac-Pilav Z, Olsson B, Nelander Set al (2019) Batch-normalization of cerebellar and medulloblastoma gene expression datasets utilizing empirically defined negative control genes. Bioinformatics 35: 3357-3364 Doi 10.1093/bioinformatics/btz066
